# The Efficacy of a Smectite-Based Mycotoxin Binder in Reducing Aflatoxin B_1_ Toxicity on Performance, Health and Histopathology of Broiler Chickens

**DOI:** 10.3390/toxins13120856

**Published:** 2021-12-01

**Authors:** Ismail Zabiulla, Venkataramaiah Malathi, H. V. L. N. Swamy, Jaya Naik, Lane Pineda, Yanming Han

**Affiliations:** 1 Poultry Science Department, Veterinary College Bangalore, Karnataka Veterinary Animal and Fisheries Sciences University, Bengaluru 560024, India; zabiullaismail01@gmail.com (I.Z.); dr.jnaik2007@rediffmail.com (J.N.); 2 Trouw Nutrition, Hyderabad 500032, India; swamy.haladi@trouwnutrition.com (H.V.L.N.S.); lane.pineda@trouwnutrition.com (L.P.); Yanming.Han@trouwnutrition.com (Y.H.)

**Keywords:** aflatoxin, bentonite, broiler chicken, mycotoxin, mycotoxin binder, smectite

## Abstract

The aim of the experiment was to investigate the efficacy of a smectite-based clay binder (Toxo-MX) in reducing the toxicological effects of aflatoxin B_1_ (AFB_1_) in commercial broiler chickens. A total of 450 one-day old male broiler chickens were randomly allocated into three treatment groups with ten replicates of 15 birds each in a 42-day feeding experiment. The dietary treatments included a negative control (NC, a basal diet with no AFB_1_ and binder), a positive control (PC, a basal diet contaminated with 500 ppb of AFB_1_) and a smectite-based mycotoxin binder(Toxo-MX, PC with smectite clay binder). AFB_1_ challenge resulted in 14 to 24% depression in growth performance, elevated levels of aspartate aminotransferase (AST), and gamma-glutamyl transferase (GGT), organ enlargement and immuno-suppression.As compared to PC, feeding of Toxo-MX improved the final weight (15%; *p* < 0.0001), average daily gain (ADG) (15%; *p* < 0.001) and feed efficiency of broilers (13%; *p* < 0.0003) but did not have any effects on liver enzyme activities. Supplementation of smectite claysignificantly increased serum globulin levels and reduced the weight of the liver (*p* < 0.05) as compared to AFB_1_-fed broiler chickens. The severity of lesions (inflammatory and degenerative changes) observed in the liver, kidney, heart, pancreas, and lymphoid organs in PC birds was reduced by feeding smectite clay. The immuno-suppression caused by AFB_1_ was moderately ameliorated in Toxo-MX groupby stimulating the production of antibodies against IBD at day 42 (*p* < 0.05). In conclusion, dietary supplementation of a smectite-based mycotoxin binder to the diet containing AFB_1_ improved growth performance, reduced toxicological effects in liver and improved humoral immune response in broilers, suggesting its protective effect against aflatoxicosis.

## 1. Introduction

Commercial broiler chickens across the globe fail to express their full genetic potential due to constant exposure to external agents such as virus, bacteria, protozoa, parasites, molds and mycotoxins. Among the various mycotoxins, aflatoxins (AF), ochratoxins, T-2 toxin, deoxynivalenol (DON), zearalenone and fumonisins are commonly detected in feeds and raw materials at considerable concentrations in different parts of the world [1]. Aflatoxins are the secondary metabolites and cancer-causing compounds produced predominantly by *Aspergillus flavus* and *A. Parasiticus* fungi [2]. Aflatoxins occur worldwide in feed and feed stuffs which results in severe economic loss to poultry and livestock industries [3]. The extent of AF contamination varies with geographic location, farming methods and the susceptibility of commodities to fungal invasion during pre-harvest, storage, and processing periods [4,5].

Numerous studies showed negative effects of AF in broiler chickens including a decrease in the efficiency of feed utilization and body weight gain, liver damage, poor immune response, and increased mortality. AF is shown to induce pathological alterations in important organs such as the liver, kidneys, and lymphoid organs [6]. Furthermore, the transmission of aflatoxin B_1_(AFB_1_) and its metabolites from feed to animal edible tissues and products, such as the liver and eggs [7], becomes particularly important as a potential hazard for human health.

Given the global economic importance of AF, many strategies have been tried to minimize their negative impact. A successful prevention strategy must be economical and capable of eliminating all traces of toxin without leaving harmful residues and should not impair the nutritional quality of the commodities [8]. Extensive research has been carried out using adsorbent (binder) materials that adsorbs to AF molecule by means of ion exchange and thereby preventing their absorption into blood circulation [9,10]. Among various binding agents, clays and yeast cell wall materials are the most tested.

Silicates are the main group of clays that are studied extensively in terms of AF binding. These include tectosilicates (zeolites), 1:1 phyllosilicates (kaolinite), 2:1 phyllosilicates (smectites, vermiculites, chlorites, micas) and sepiolite. All silicates, however, are not the same in terms of their ability to bind AF and among the above, smectites have shown greater binding efficacy against AF. The ability of smectite clays to bind mycotoxins depends on pH in the gut, molecular arrangements, and its geographic region of origin [11]. Smectite clays possess high AF adsorption capacity due to its high surface area, ion exchange capacity, and ability to swell in the presence of water [12], and the efficacy has been proven in vivo in broiler chickens [13]. The leading hypothesis on the bonding mechanism between adsorbed aflatoxins and smectites is the electron donor–acceptor (EDA) model. Other models such as selective chemisorption, H-bonding, and bonding through furan rings were proposed.

The present research was conducted to evaluate the effects of smectite clay, on various performance, organ morphology, serum biochemistry and histopathology parameters in broiler chickens exposed to AFB_1_.

## 2. Results

### 2.1. Performance Parameters

Data on the average daily gain (ADG), the average daily feed intake (ADFI) and the feed conversion ratio (FCR) areshown in Table 1. The ADG was significantly (*p* < 0.05) lowered by AFB_1_ in all the three phases of growth (d 1–14, 15–28 and 29–42). Feeding smectite binder along with AFB_1_ did not show significant improvement in ADG during d 1–14; however, in the succeeding phases of growth and overall, from d1–42, ADG was significantly (*p* < 0.05) increased. AF challenge significantly (*p* < 0.05) reduced the ADFI in all the phases except 15 to 28 d period. A feeding binder did not improve ADFI in any phase. FCR in all the phases and during 1 to 42 d was significantly (*p* < 0.05) higher in the AF challenged groups. Feeding smectite clay significantly improved the FCR in all the phases as compared to AF fed birds and it was comparable to birds in Control group.

### 2.2. Serum Biochemistry

Serum concentrations of various liver enzymes, bilirubin, and proteins are indicated in Table 2. Serum concentrations of aspartate aminotransferase (AST) and gamma-glutamyl transferase (GGT) increased significantly (*p* < 0.05) in AF challenged groups, indicating considerable liver damage. Supplementation of smectite clay did not significantly reduce AST levels in the serum. Though not significant, huge numerical differences in the lactate dehydrogenase (LDH) concentrations were noticed among different groups. Serum bilirubin concentrations (total and direct), indicative of liver damage, did not significantly increase with AF challenge. Total protein, albumin and globulin levels in serum were not significantly altered by AF challenge but there was a tendency towards lowered globulin concentrations. Supplementation of smectite clay to the AF group significantly altered the albumin and globulin concentration in the serum.

### 2.3. Antibody Titres

Antibody titres against Newcastle disease (ND) were not significantly affected either by AF challenge or supplementation of binder (Table 3). The antibody response to infectious bursal disease (IBD) vaccination, both on d 21 and 42, was significantly reduced in AF-fed chickens suggesting an immunosuppressive effect of the toxin. The titers against IBD vaccine were moderately improved by the supplementation of smectite clay to the toxin group on d 42.

### 2.4. Dressing Yield and Organ Weights

The dressing yield, both on d 21 and 42, was significantly (*p* < 0.05) lower in AF-challenged group as compared to the control but there was no improvement by the supplementation of smectite clay binder. Significant differences were noticed in the relative weights of the liver, kidney, heart, spleen, and pancreas among the groups (Table 4). The weights of the liver and kidney were significantly more in AF-challenged groups both on d 21 and 42, which is a typical of aflatoxicosis. Smectite clay feeding did not alter the weights of the liver and kidney on d 21 and kidney on d 42. While on d 42, the weight of the liver significantly reduced upon the feeding binder to AF-challenged chicken. The weight of the heart on d 21 increased in the toxin group which was partly reduced by feeding the binder. On d 21, AF caused higher spleen weights, which was not reduced by the addition of smectite clay. No significant differences in weights of the bursa of Fabricius and thymus were noticed among different groups. Significantly heavier pancreas was observed in the toxin group both on d 21 and 42, while the binder was able to partly reduce weights at d 21. No difference was seen among the three groups for abdominal fat content on 21st day, while on 42nd day, less abdominal fat was recorded in the group fed AFand the binder as compared to control group.

### 2.5. Histopathology

Histopathological examination of the visceral organs in the birds of NC diet revealed normal architecture of the liver, kidney, heart, spleen, bursa, thymus, and pancreas on 21 d and 42 of the experiment. The same examination in AF group revealed severe inflammation, congestion, and degenerative changes. Most of these changes were prevented by the addition of smectite binder to AF group.

#### 2.5.1. Liver

**Figure 1:**
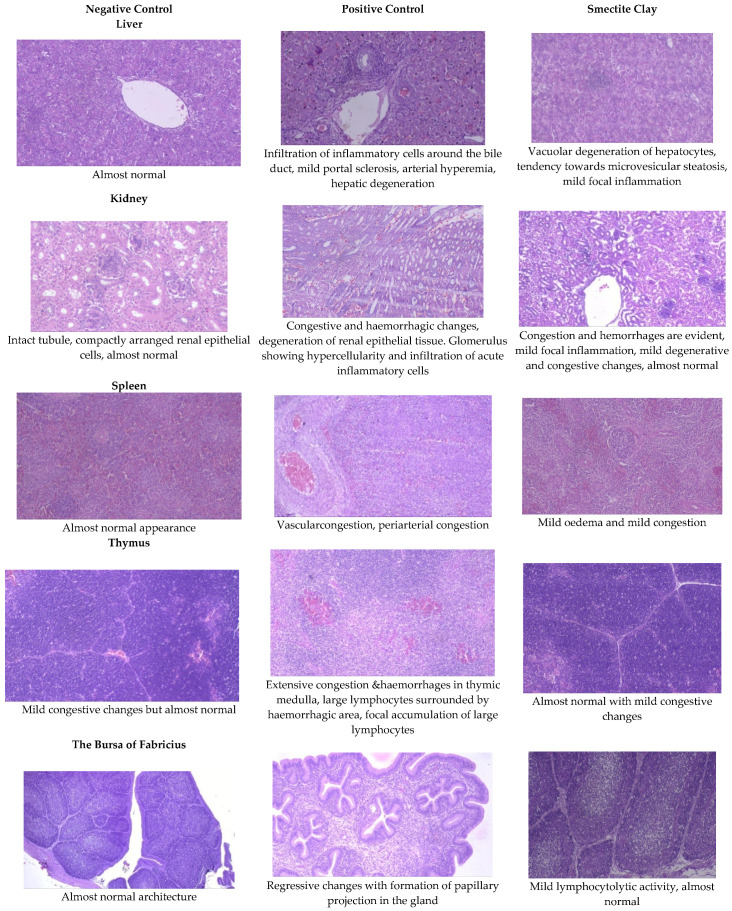

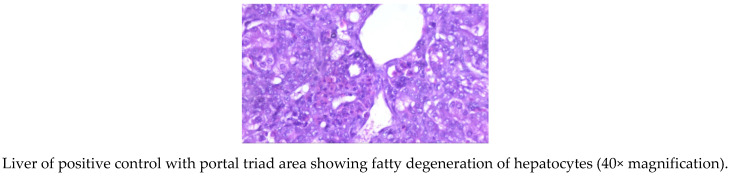
Effect of smectite clay on histopathology of aflatoxin-challenged broiler chickens on d 21 (haematoxylinand eosin stain, 10× magnification).

**Figure 2:**
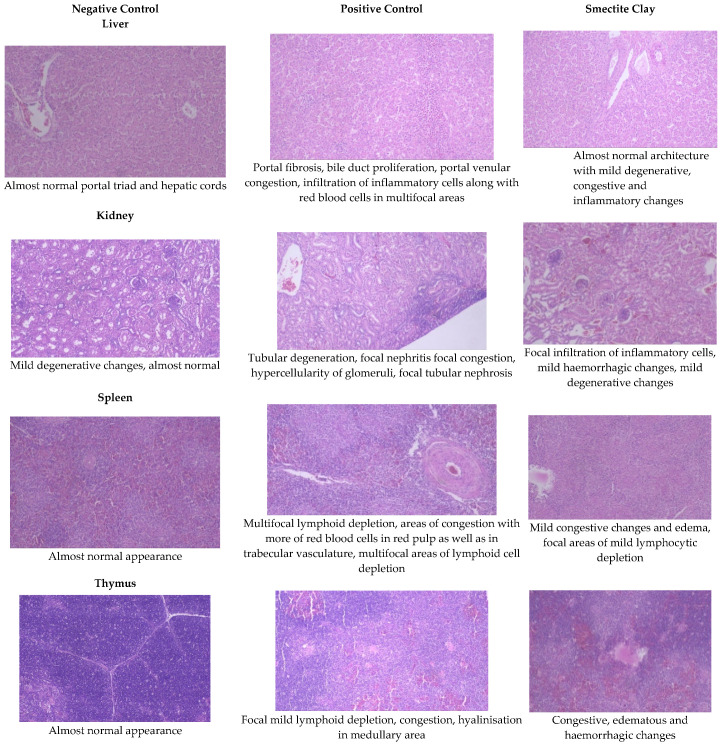

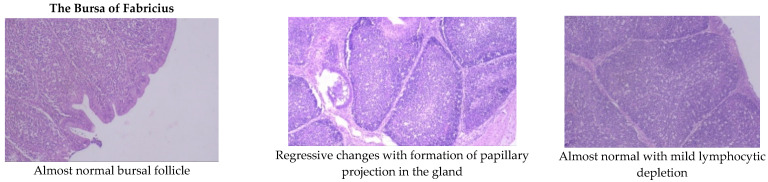
Effect of smectite clay on histopathology of aflatoxin-challenged broiler chickens on d 42 (Haematoxylin& eosin stain, 10× magnification).

The livers of AF fed birds revealed portal hepatitis, mild portal congestion, sinusoidal congestion, biliary hyperplasia, cellular swelling, and cytoplasmic vacuolar degeneration of hepatocytes. Portal arterial hyperemia, infiltration of inflammatory cells around periductular and perivascular region and mild portal sclerosis were observed in 21-day old birds (Figure 1). In 42-dayold birds, these changes progressed in severity followed by varying degrees of vacuolar degeneration of hepatocytes with multifocal infiltration of mononuclear cells, portal fibrosis, bile duct proliferation, portal vascular congestion and sinusoidal congestion in several areas along with microvesicular steatosis (Figure 2).

Birds fed a binder diet showed similar changes, but of a reduced magnitude and severity. The changes observed were more of a degenerative type rather than necrosis. Almost normal architecture with mild degenerative, congestive, and inflammatory changes were noticed in the binder group (Figure 1 and Figure 2).

#### 2.5.2. Kidneys

The histopathological lesions observed in 21-day-old toxin fed birds included vascular congestion, focal hemorrhages, and multifocal cortical tubular epithelial degeneration along with glomerulitis and hypercellularity (Figure 1). On d 42, there were multifocal hemorrhages, focal infiltration of inflammatory cells, multifocal areas of tubular epithelial degeneration and glomerular hypercellularity (Figure 2).

On d 21 and 42, kidneys of chicken fed smectite binder revealed a mild degree of congestion, focal infiltration of inflammatory cells, and an almost intact lining of renal epithelial cells with a very mild degree of degenerative changes. The maintenance of almost normal architecture of convoluted tubules with lining epithelium showing negligible degeneration and interstitial inflammation was observed on both d 21 and 42 birds (Figure 1 and Figure 2).

#### 2.5.3. Heart

On d 21, the heart tissue of birds in the toxin group showed mild myocarditis, myocardial congestion and hemorrhages, and mild pericardial edema. At the end of the experiment (d 42), cardiac musculature of the birds revealed a mild-to-moderate degree of hemorrhages and degenerative changes, mild perivascular fibrosis, edema and congestion, focal inflammations, and the hypertrophy of ventricular valves along with the narrowing of lumen of ventricles, with the occasional retention of the blood.

Birds supplemented with smectite clay showed marked improvement and revealed mild focal myocarditis, mild vascular congestion, a mild degree of myocardial and pericardial edema during the 21st and 42nd days of the experiment.

#### 2.5.4. Spleen

The spleen of AF fed birds revealed multifocal lymphoid depletion along with vascular congestion on d 21 of the experiment and continued untilthe end of the experiment along with multifocal secondary follicle formation, proliferation of reticular tissue, perivascular fibrosis, and congestion in red pulp (Figure 1 and Figure 2). The smectite clay supplemented group showed mild congestive changes, edema, and lymphocytic depletion.

#### 2.5.5. Pancreas

Aflatoxin challenged birds revealed focal pancreatitis, and acini surrounded by inflammatory cells on 21st day of experiment in several areas and continued till the end of the experiment. There were also focal exocrine pancreatitis and necrosis, and congestionin pancreatic islets. Binder supplemented chickens showed an almost normal appearance of the pancreas on d 21 but a mild degree of vascular congestion on the 42nd day of the trial.

#### 2.5.6. Thymus

Mild focal lymphoid depletion, focal hemorrhages, and occasional hyaline deposits were observed on the 21st day of the trial in PC birds (Figure 1). At the end of the trial, a starry sky appearance characterized by medullary necrosis with compactly arranged cortical lymphoid cells, becoming gradually less in number towards medullary area, was noticed (Figure 2). Feeding binder to AF challenged birds had an almost normal architecture on the 21st day but at end of the trial, there were mild congestive, edematous, and hemorrhagic changes (Figure 1 and Figure 2).

#### 2.5.7. The Bursa of Fabricius

Aflatoxin fed birds showed mild lymphoid cell depletion and edema on the 21st day of the trial. These lesions were present till the end of the experiment along with regressive changes and formation of papillary projections in the gland (Figure 1 and Figure 2). Feeding smectite clay showed mild lympholytic activity on the 21st day while the atrophy of follicles was observed on day 42.

## 3. Discussion

Body weight gain was depressed in AF fed birds. Supplementing 0.2% of smectite clay binder counteracted the toxic effects and improved weight gain. Similar responseswere reported by Kumar et al., (2014) and Yalgod (2014) [14,15]. The reduced feed intake was observed in toxin fed birds and this agrees with Vekiru et al., (2015) and Mendieta et al., (2018) [16,17]. FCR of AF fed birds was significantly higher in comparison with that of control groups. The increased FCR is attributed to the inhibition of protein, enzymes and lipid synthesis, and liver damage caused by AF [15,18,19,20,21]. Nelson et al., (1982) showed that AF reduces the ability of birds to digest dry matter and utilize amino acids and energy from an AF-contaminated diet [22]. In this study, the dietary inclusion of smectite clay improved the FCR of AF fed birds indicating the role of binder in amelioration of toxic effects of AF which is in accordance with the finding of Desheng et al., (2005) and Bailey et al., (2006) [23,24].

Elevated levels of serum AST and GGT were observed in birds receiving 0.5 ppm of AF as compared to control group chickens, indicating considerable damage to the liver. Similar findings of elevated activities of serum enzymes due to aflatoxicosis were observed by many researchers [25,26,27]). Raju and Devegowda (2000) recorded no difference in AST levels in birds fed AF up to 0.3 ppm for 21 days [25]. Broilers fed with 1 ppm AFB_1_ for 42 days of age showed no effect on AST activity [28]. Such a variation in serum enzyme activities is influenced by various factors, namely, the concentration and duration of exposure toAF, the strain and sex of the birds, health, and nutritional status, as well as other environmental factors. The discrepancies in enzyme profile as revealed by several researchers depict that the enzyme levels may not suggest the extent of liver damage or could not be a true indicator during aflatoxicosis [29].

In this experiment, there was a moderate reduction in the serum globulin concentration in AF challenged birds as compared to the control group. Similar findings were reported in broilers fed AFB_1_ ranging from 0.3 to 5 ppm by other researchers as well [30]. The hypoproteinemia observed in AF fed birds is primarily due to the decrease in feed consumption and the inactivation of the biosynthetic pathway of enzymes for protein synthesis [31]. The dietary inclusion of smectite clay counteracted these effects in a significant manner. This indicated that the reduced damage to the liver in the binder fed group is due to the adsorption of toxins in the gut, rendering it unavailable for absorption. Improved serum levels of globulin in AF-challenged broilers supplemented with smectite clay was also reported by Rosa et al., (2001); Shi et al., (2006) and Denli et al., (2009) [10,19,28].

Reduced antibody titres against IBD were observed in birds receiving 0.5 ppm of AF and these results concurred with earlier findings of Jahanian et al., (2019) and Yalgod (2014) in chickens fed with AF for 42 d [15,32]. A fall in the antibody titres against IBD upon feeding AF is attributed to the regression of the bursa of Fabricius, lymphocytolysis and lymphoid depletion. Aflatoxin in the diet increases the specific activity of lysosomal enzymes in the liver and muscles causing enhanced degradation of antibodies [33].Incorporation of the binder moderately improved antibody titres against IBD on d 42 and this may suggest protective effect against aflatoxicosis.

A decrease in dressing percentage was observed on both d 21 and 42 of the experiment in AF-challenged chickens. The findings of the present study agree with Pasha et al., (2007) [34]. Birds receiving AF did not show any significant differences in the abdominal fat content on d 21 of the study. While, on 42nd day, the addition of binder to AF showed a decrease in the percentage of abdominal fat as compared to birds fed the control diet. This may be considered as a non-specific benefit of smectite binder.

Increased relative weights of the liver and kidneys in comparison to the control group in this study are in accordance with earlier research [25,35,36,37,38]. The increase in the relative weight of the liver in AF-treated birds could be attributed to AF-induced impaired fat metabolism in the liver with an increase in the fat content of the hepatocytes [39]. Inhibition of phospholipids and cholesterol synthesis can lead to hepatic lipidosis, which in turn affects the transportation of lipids from the liver [40]. In addition, degenerative, inflammatory, and vascular changes caused by AF in visceral organs might be responsible for increased weights and this observation is upheld by the histopathological lesions in liver showing severe fatty changes and lipidosis. Higher weights of kidneys in AF challenged chicken seen in this study could be due to the development of vacuolar degeneration and renal damage during aflatoxicosis [15,41].

The increase in the relative weight of the heart can be correlated to the earlier findings of enlargement of the heart in AF-fed broiler birds [42,43,44]. The increase in the relative weights of the pancreas could be due to the damage to the pancreas as evident by histopathological observations characterized by severe vascular congestion and the infiltration of mononuclear cells, which is further supported by the findings of Valchev et al., (2014) [45].The increase in the relative weight of the spleen as observed in this study is in accordance with Shi et al., (2006) and Hussain and Khan (2008) [19,36].The weights of thymus and bursa were not significantly affected by AF in this study, and these contrast with the findings of Santhosh Kumar (2003) [41]. The significant reduction in relative weights of the liver, and a partial reduction in the relative weights of the heart and pancreas by the addition of binder to the AF-contaminated diet indicates potential organ protection of the binder in question.

Histopathological observations in visceral organs clearly depicted the toxic effect of AF. The effects of AF on the histology of the liver observed in the current study agree with several previous research work [17,41,46,47,48,49,50,51,52]. Aflatoxin causes disturbances in lipid, carbohydrate, and protein metabolism [53,54], as well as haematopoiesis [55]. Multifocal and multiple varying sized cytoplasmic vacuolation with perilobular location in AF-fed birds have also been reported in other studies [56]. The dilation of arterioles causes congestion, and this may be due to an increased flow of blood to the tissue [57]. Slow metabolic activity within the cell leads to the accumulation of metabolic products which brings about theenlargement and swelling of hepatocytes. Aflatoxins are cytotoxic and inhibits the proliferation of hepatocytes [58]. The vacuolar degeneration of hepatocytes may be due to impaired lipid transport [59]. The observed hyperplasia of the bile duct epithelium may be due to the direct effect of AF on biliary epithelial cells or excessive production of prostaglandins [60,61]. Smectite clay supplementation to AF challenged birds caused asignificant reduction in the magnitude and severity of hepatic lesions indicating its protective role.

The histological findings in the kidney observed in this study are well supported by the observations of previous workers [15,62]. AF is eliminated mainly through the kidneys and the accumulation of a high concentration of toxins impairs excretory function and leads to congestion with patho-morphological alterations [63]. Aflatoxin induced nephrotoxicity is thought to be due to the interference with transport function in collecting tubular cells together with the diffused impairment of the proximal tubular function [47]. Aflatoxins and their metabolites exhibit their toxic effect on different parts of nephrons before being excreted, resulting in nephrotoxicity [64]. The supplementation of smectite clay to AF-challenged birds noticeably reduced the severity of lesions and this can be attributed to clay binding to AF irreversibly.

Mild myocarditis, endothelial and myocardial hemorrhages, pericardial edema, myocardial infiltration of inflammatory cells and congestive changes were observed microscopically for the entire period of study in AF-fed birds, which matched with earlier reports [15,62]. In birds fed with both AF and the binder, the heart remained morphologically normal and could be comparable with the control group. Focal exocrine pancreatitis, focal congestion, and mild necrosis of islet cells and acinar cells were observed in the pancreas of birds fed AF and these correspond to findings from earlier reports [45,65]. Feeding smectite clay to the toxin fed birds effectively alleviated the severity of AF-induced histological lesions in the pancreas.

Aflatoxin-induced changes in the histology of lymphoid organs in this study, namely the thymus, spleen, and the bursa of Fabricius are in line with Ortatatli and Oguz (2001) and Mendieta et al., (2018) [17,66]. Similarly, Sur and Celik (2003) also observed depletion of lymphoid cells and regression of lymphoid organs following experimental aflatoxicosis in broilers [67]. Aflatoxins in the diet of chicken resulted in destruction of thymic cortex, degeneration of follicles in bursa of Fabricius and decrease of splenic T cells [68]. The inclusion of smectite clay in the diet could significantly ameliorate most of the adverse effects of AF which is indicative of the protective role of the binder against aflatoxicosis.

A slightly higher AF level was used here (500 ppb) to producesignificant differences from the control group. In a field situation where commercial poultry feeds will have AF levels 4 to 5 timeslower than this, a complete reversal of the negative effects is possible at 0.2% clay in the feed.

In conclusion, afaltoxin challenge in broiler chicken was effective at 0.5 ppm level in feed with significant drop in weight gain, feed efficiency, humoral immune response, and assault to most of the visceral organs as evidenced by the serum biochemical parameters and histopathological observations. The supplementation of smectite clay at 0.2% in feed to aflatoxin challenged broilers considerably reduced the magnitude of toxic effects of aflatoxin and improved growth and immune response. Hence, smectite clay could be successively used in feed to ameliorate the toxic effects of aflatoxins in broiler chickens.

## 4. Materials and Methods

### 4.1. Aflatoxin B_1_

The required quantity of aflatoxin B1 (AFB_1_) was produced by solid substrate fermentationas per the method of Shotwell et al., (1996) [69]. *Aspergillus parasiticus* culture (MTCC 2797) was obtained from Microbial Type Collection and Gene Bank, CSIR—Institute of Microbial Technology, Chandigarh, India. AFB_1_ content of the culture material was determined by thin-layer chromatography by the method of the Association of Official Analytical Chemists (1995) [70], followed by HPLC. The aflatoxin content in the powdered substrate consisted of 84.32% AFB_1_, 9.33% AFB_2_, 3.87% AFG_1_, and 2.48% AFG_2_.

### 4.2. Experimental Birds and Diets

A total of 450 male day-old commercial broilers were divided randomly into 30 groups of 15 chicks. Each tensuch groups were allotted to one of the three dietary treatments. Each replicate group of chicks was housed in an independent pen in an open sided deep litter house and reared under uniform standard conditions throughout the study with ad libitum feed and water and a 24-h light schedule. The experimental protocol was approved by the Institutional Animal Ethical Committee (IAEC) at the University (protocol code V0010510_MX and date of approval 18 November 2017).

A basal diet (NC) was formulated using corn and soybean meal to contain the nutrients according to the specifications of NRC 1994 [71] (Table 5). To the basal diet, powdered culture containing known concentration of AFB_1_ was incorporated to yield 0.5 ppm AFB_1_ (PC). Smectite clay (Toxo-MX, 0.2%) was added to the toxin contaminated feed to make it as treatment 3. The smectite clay contained 94% dioctahedral smectite, 3% sodium feldspar, 1% each of illite and maghemite, and traces of quartz and calcite, as assessed by an X-ray diffraction study. Chicks were fed with a pre-starter diet from 1 to 14 days, a starter diet from 15 to 28 days and a finisher diet from 29 to 42 days of age (Table 1). Compounded experimental diets of all the groups were analyzed for AFB_1_ content to counter check the required levels.

### 4.3. Data Collection

#### 4.3.1. Performance

All chicks were weighed individually every week. Dataon weekly feed intake, feed conversion ratio (FCR-feed intake/weight gain) and mortality wererecorded in each replicate group.

#### 4.3.2. Serum Biochemistry

On the 42nd d of age, 2 mL blood was collected into plain non-heparinized tubes from 2 birds from each pen from the wing vein. Serum was collected and stored at −40 °C. Serum samples were analyzed individually for bilirubin, total protein, albumin and globulin and the activities of aspartate transaminase (AST), alkaline phosphatase (ALP), gamma-glutamyl transferase (GGT) and lactate dehydrogenase (LDH) using a serum biochemical analyzer as per the recommendations of the manufacturer’s kit.

#### 4.3.3. Immune Competence

The serum collected on days 21 and 42 was subjected to antibody titer assays against the Newcastle disease virus and infectious bursal disease virus. The antibody titer against the Newcastle disease virus was carried out by haemagglutination followed by a haemagglutinationinhibition test. The micro-test method as described by Allan and Gouch (1974) was used for the detection of HI titres from the serum samples. The antibody titer against IBDV was measured using Poultry Diagnostic and Research Center (PDRC) indirect ELISA kit and performedas per the methodology recommended by the manufacturer.

#### 4.3.4. Dressing Yield and Organ Weights

On day 21 and 42, the same two birds from each pen from which the blood samples were collected, were immediately slaughtered and the weights of the liver, heart, spleen, kidney, bursa of fabricius, thymus, pancreas and abdominal fat were recorded. The percentage of the live animal that ends up as a carcass after defeathering and the removal of offal was calculated and recorded.

#### 4.3.5. Histopathology

Immediately after recording the weights of visceral organs, portion of liver, heart, spleen, kidney, bursa of Fabricius, thymus and pancreaswere collected into glass bottles and fixed with 10% neutral buffered formalin solution for histopathological studies. Paraffin embedded tissues were sectioned to 5–6 μm thickness and stained with haematoxylin and eosin following standard procedures [72].

### 4.4. Statistical Analysis

Pen was considered as the experimental unit for statistical analysis. The data were subjected to one-way ANOVA using SPSS version 20. Means were compared by Tukey’s multiple comparison test at *p* ≤ 0.05.

## Figures and Tables

**Table 1:** Effect of smectite clay on growth parameters of aflatoxin-challenged broiler chickens.

Parameters	Control ^1^	PC ^1^	Toxo-MX ^1^	SEM
Day 1–14				
ADG ^2^, g	20.03 ^a^	16.51 ^b^	17.85 ^b^	0.365
ADFI ^2^, g	32.69 ^a^	30.42 ^b^	29.10 ^b^	0.524
FCR ^2^	1.637 ^b^	1.847 ^a^	1.633 ^b^	0.030
Day 15–28				
ADG, g	60.69 ^a^	43.06 ^c^	50.74 ^b^	1.478
ADFI, g	111.72	109.41	103.71	2.195
FCR	1.852 ^b^	2.550 ^a^	2.046 ^b^	0.074
Day 29–42				
ADG, g	86.53 ^a^	65.81 ^c^	77.21 ^b^	1.716
ADFI, g	156.81 ^a^	134.98 ^b^	143.27 ^b^	2.623
FCR	1.815 ^b^	2.057 ^a^	1.855 ^b^	0.034
Day 1–42				
ADG, g	55.98 ^a^	41.89 ^c^	48.27 ^b^	1.120
ADFI, g	104.78 ^a^	97.04 ^b^	96.85 ^b^	1.398
FCR	1.870 ^b^	2.320 ^a^	2.012 ^b^	0.045

^1^ Control = no aflatoxin challenge and smectite use; PC = 500ppb aflatoxin challenge; Toxo-MX = 500 ppb aflatoxin challenge with 0.2% smectite clay, ^2^ ADG: average daily gain; ADFI: average daily feed intake; FCR: feed conversion ratio; SEM: standard error of means. ^a–c^ Means within a row bearing different superscripts differ significantly (*p* ≤ 0.05).

**Table 2:** Effect of smectite clay on serum biochemical parameters of aflatoxin-challenged broiler chickens.

Groups	AST ^2^(U/L)	GGT ^2^ (U/L)	LDH ^2^ (U/L)	Total Bilirubin (mg/dL)	Direct Bilirubin (mg/dL)	Total Protein (g/dL)	Albumin (g/dL)	Globulin (g/dL)
NC ^1^	172.15 ^b^	8.62 ^b^	221.25	0.34	0.16	3.53	2.31 ^a^	1.21 ^ab^
PC ^1^	248.86 ^a^	16.08 ^a^	592.34	0.18	0.12	2.99	2.13 ^a^	0.86 ^b^
Toxo-MX ^1^	242.50 ^a^	13.03 ^a^	407.03	0.73	0.34	3.04	1.30 ^b^	1.74 ^a^
Pooled SEM	9.82	0.77	67.23	0.11	0.05	0.12	0.11	0.11

^1^ Control = no aflatoxin challenge and smectite use; PC = 500 ppb aflatoxin challenge; Toxo-MX = 500 ppb aflatoxin challenge with 0.2% smectite clay, ^2^ AST = aspartate amino transferase; GGT =γ-glutamyl transferase; LDH = lactate dehydrogenase; SEM = standard error of means. ^a,b^ Means within a row bearing different superscripts differ significantly (*p* ≤ 0.05).

**Table 3:** Effect of smectite clay onantibody titres against ND and IBD ofaflatoxin-challenged broiler chickens.

Groups	Day 21		Day 42	
	Log_10_ND ^2^ Titres	IBD ^2^ Titres	Log_10_ND Titres	IBD Titres
NC ^1^	1.14	2159 ^a^	1.34	1678 ^a^
PC ^1^	1.15	1723 ^b^	1.09	1182 ^b^
Toxo-MX ^1^	1.36	1697 ^b^	1.16	1479 ^ab^
Pooled SEM	0.04	49.21	0.05	61.17

^1^ Control = no aflatoxin challenge and smectite use; PC = 500ppb aflatoxin challenge; Toxo-MX = 500 ppb aflatoxin challenge with 0.2% smectite clay. ^2^ ND = Newcastle disease; IBD = infectious bursal disease; SEM = standard error of means. ^a,b^ Means within a row bearing different superscripts differ significantly (*p* ≤ 0.05).

**Table 4:** Effect of smectite clay on dressing yield, organ weights and abdominal fat, as % of body weightofaflatoxin-challenged broiler chickens.

Groups	Dressing %	Liver	Kidney	Heart	Spleen	Bursa	Thymus	Pancreas	Abdominal Fat
Day 21									
NC ^1^	67.92 ^a^	2.48 ^b^	0.42 ^b^	0.64 ^b^	0.09 ^b^	0.24	0.28	0.34 ^b^	0.82
PC ^1^	64.92 ^b^	3.23 ^a^	0.59 ^a^	0.71 ^a^	0.14 ^a^	0.19	0.26	0.40 ^a^	0.69
Toxo-MX ^1^	65.13 ^b^	3.05 ^a^	0.64 ^a^	0.69 ^ab^	0.13 ^a^	00.19	0.25	0.37 ^ab^	0.68
SEM	0.23	0.05	0.02	0.01	0.005	0.01	001	0.01	0.03
Day 42									
NC	73.95 ^a^	1.99 ^c^	0.31 ^b^	0.51	0.12 ^b^	0.07	0.37	0.20 ^b^	1.19 ^a^
PC	69.04 ^b^	3.27 ^a^	0.58 ^a^	0.54	0.16 ^ab^	00.09	0.32	0.28 ^a^	0.99 ^ab^
MX	69.62 ^b^	2.75 ^b^	0.55 ^a^	0.56	0.22 ^a^	00.07	0.35	0.25 ^a^	0.89 ^b^
SEM	0.51	0.08	0.02	0.01	0.01	0.01	0.01	0.01	0.04

^1^ Control = no aflatoxin challenge and smectite use; PC = 500 ppb aflatoxin challenge; Toxo-MX = 500 ppb aflatoxin challenge with 0.2% smectite clay; SEM = standard error of means. ^a–c^ Means within a row bearing different superscripts differ significantly (*p* ≤ 0.05).

**Table 5:** Ingredient composition (kg/100 kg of feed) of the experimental diets.

Ingredients	Pre-Starter Diet (1–14 Days)	Starter Diet (15–28 Days)	Finisher Diet (29–42 Days)
Corn	53.47	57.00	60.47
Soybeanmeal	41.0	35.77	31.00
Vegetable oil	2.2	4.0	5.5
* Mineral mixture	1.5	1.5	1.5
Dicalciumphosphate	1.0	0.9	0.8
Common Salt	0.3	0.3	0.3
** Vitaminpremix	0.2	0.2	0.15
DL-Methionine	0.2	0.2	0.18
B-complex	0.1	0.1	0.1
Antibiotic ***	0.03	0.03	-
**Total**	**100**	**100**	**100**
Analysed values			
Moisture	10.22	10.12	9.43
Crude protein	22.68	20.81	19.31
Crude fat	4.53	5.63	7.92
Crude fiber	4.71	4.69	4.51
Ash	5.7	5.9	7.6
Ca	1.02	0.99	0.96
*p*	0.459	0.454	0.422
Aflatoxin B_1_ in control diet	Traces	Traces	Traces
Aflatoxin B_1_ in PC and Toxo-MX diet	456 ppb	425 ppb	484 ppb

* MineralMixture: each 100 g contains: magnesium oxide—1.48 g, ferrous sulphate—6.0 g, copper sulphate—0.05 g, manganese sulphate—0.04 g, potassium iodide—0.001 g, zinc sulphate—1.0 g, potassium chloride—17.09 g and sodium selenate—0.001 g. ** Vitamin premix: each 100 g contains vitamin AD3 (vitamin A—1,000,000 IU/g, vitamin D—200,000 IU/g)—0.165 g, vitamin K3—0.103 g, vitamin E—2.4 g, thiamine mononitrate—0.206 g, riboflavin—0.513 g, pyridoxine hydrochloride—0.309 g, cyanocobalamin—0.00031 g, folic acid—0.103 g, niacin—4.124 g, Ca-D–pantothenate—1.031 g, biotin—1.5 g, maltodextrin—89.545 g. *** Oxytetracycline.
